# Persistent accelerated epigenetic ageing in a longitudinal cohort of vertically infected HIV-positive adolescents

**DOI:** 10.1007/s13365-023-01130-6

**Published:** 2023-05-13

**Authors:** Sarah J. Heany, Andrew J. Levine, Maia Lesosky, Nicole Phillips, Jean-Paul Fouche, Landon Myer, Heather J. Zar, Dan J. Stein, Steve Horvath, Jacqueline Hoare

**Affiliations:** 1 SA MRC Unit On Risk & Resilience in Mental Disorders, Department of Psychiatry and Mental Health, University of Cape Town, Cape Town, South Africa; 2 Neuroscience Institute, University of Cape Town, Cape Town, South Africa; 3 Department of Neurology, David Geffen School of Medicineat the , University of California, Los Angeles, Los Angeles, CA USA; 4 Division of Epidemiology and Biostatistics, School of Public Health & Family Medicine, University of Cape Town, Cape Town, South Africa; 5 Centre for Infectious Disease Epidemiology and Research, Division of Epidemiology and Biostatistics, School of Public Health & Family Medicine, University of Cape Town, Cape Town, South Africa; 6 Department of Paediatrics and Child Health, Red Cross War Memorial Children’s Hospital, Cape Town, South Africa; 7 Medical Research Council Unit On Child and Adolescent Health, University of Cape Town, Cape Town, South Africa; 8 Department of Human Genetics, David Geffen School of Medicine, University of California, Los Angeles, Los Angeles, CA USA; 9 Department of Biostatistics, School of Public Health, University of California, Los Angeles, Los Angeles, CA USA

**Keywords:** Accelerated ageing, DNA methylation, Epigenetic clock, Perinatal HIV

## Abstract

We have previously shown accelerated ageing in adolescents perinatally infected with HIV (PHIV +), based on discrepancies between epigenetic and chronological age. The current study examines follow-up longitudinal patterns of epigenetic ageing and the association of epigenetic ageing with cognition as well as whole brain structure changes in PHIV + and healthy controls enrolled in the Cape Town Adolescent Antiretroviral Cohort Study (CTAAC). The Illumina EPIC array was used to generate blood DNA methylation data from 60 PHIV + adolescents and 36 age-matched controls aged 9–12 years old at baseline and again at a 36-month follow-up. Epigenetic clock software estimated two measures of epigenetic age acceleration: extrinsic epigenetic accelerated ageing (EEAA) and age acceleration difference (AAD) at both time points. At follow-up, each participant completed neuropsychological testing, structural magnetic resonance imaging, and diffusion tensor imaging. At follow-up, PHIV infection remains associated with increased EEAA and AAD. Accelerated epigenetic ageing remained positively associated with viral load and negatively associated with CD4 ratio. EEAA was positively associated with whole brain grey matter volume and alterations in whole brain white matter integrity. AAD and EEAA were not associated with cognitive function within the PHIV + group. Measures of epigenetic ageing, as detected in DNA methylation patterns, remain increased in PHIV + adolescents across a 36-month period. Associations between epigenetic ageing measures, viral biomarkers, and alterations in brain micro- and macrostructure also persist at 36-month follow-up. Further study should determine if epigenetic age acceleration is associated with cognitive functional changes due to brain alterations in later life.

## Introduction

Human immunodeficiency virus (HIV) infection presents a significant public health burden in many countries, with 360,000 adolescents between 10 and 19 years living with HIV in South Africa alone. As the reach of medical interventions has increased, more children perinatally infected with HIV survive into adolescence and adulthood. Throughout their development, perinatally infected HIV-positive adolescents (PHIV +) face unique challenges to their neurodevelopment with HIV disease progression being linked to changes in myelination and inflammation in the developing brain (Hoare et al. 2015a, b), as well as decreased gyrification and smaller cortical volumes and surface area in PHIV + (Hoare et al. 2018).

Epigenetic ageing measures have recently been developed and refined (Hannum et al. 2013; Horvath 2013; Levine et al. 2018) and detect age-related changes in DNA methylation levels specifically across CpG sites. Differences between chronological and epigenetic ages can indicate accelerated tissue ageing. Conditions including cognitive decline (Marioni et al. 2015a), Alzheimer’s severity (Levine et al. 2015a), obesity (Horvath et al. 2014), cancer risk (Levine et al. 2015b; Dugue et al. 2018), and all-cause mortality (Marioni et al. 2015b) have been associated with increased epigenetic ageing.

Our recent research has shown that DNA methylation–related ageing is increased in PHIV + adolescents compared to healthy controls (HC) (Horvath et al. 2018; Levine et al. 2018) and that epigenetic ageing in both PHIV + and HCs is associated with altered neuronal microstructure in many grey and white matter regions across the brain (Hoare et al. 2020, 2021). Accelerated epigenetic ageing was also associated with decreased cognitive performance in the domains of attention, executive function, working memory, and processing speed, and these results were more significant in the PHIV + group. Additionally, age acceleration was associated with higher viral load (VL) and lower CD4 count. Epigenetic ageing was also higher in PHIV + on second- and third-line antiretroviral treatment (ART), compared to those on first-line ART.

Other groups have also detected altered epigenetic ageing in HIV + adults (Nelson et al. 2017) and have linked a lower CD4/CD8 T cell ratio to both methylation-based epigenetic ageing and HIV progression (Gross et al. 2016). Shiau et al. (2021b) detected that EEAA was higher in HIV + American adults, and was also associated with detectable VL, and was negatively associated with executive function, attention, and language performance. One recent study on epigenetic ageing in North American PHIV + youth found that it was predictably associated with VL and CD4 counts (Shiau et al. 2021a). There is a paucity, however, of insights in adolescent PHIV + cohorts generally that we hope to address with our longitudinal study.

Although neurodevelopment and cognition have been studied in adolescents with HIV, longitudinal data on epigenetic data and their associations with white and grey matter neuroimaging and cognition in youth are lacking. Here, we assess epigenetic ageing progression in relation to perinatal HIV infection at 36-month follow-up in the Cape Town Adolescent Antiretroviral Cohort (CTAAC), as well as the relationships of epigenetic ageing with white and grey matter structure and cognitive functioning. We also consider the role of health and social factors such as CD4 ratio, VL, and education level. We hypothesised that epigenetic ageing would continue to be more significant in PHIV + compared to HC and would be associated with higher VL, lower CD4 cell ratio, failing first-line ART, altered neuroimaging results, and cognitive impairment.

## Materials and methods

### Participants

Participants were previously recruited from four ART clinics to CTAAC for baseline data collection when they were aged between 9 and 12 years. For further information on the recruitment process, see Heany et al. (2020). Two hundred four patients and 48 controls were assigned to the CTAAC neuro sub-study at baseline. Demographically matched controls from the same communities were recruited and were confirmed to be HIV negative. At 36-month follow-up, 125 patients and 40 controls were retained in the study. Blood samples from 60 randomly selected patients and 36 randomly selected controls were sent for DNA methylation analysis. Only this subsample was sent for methylation analysis due to budget constraints.

### Neurocognitive functioning

Each participant completed a comprehensive neuropsychological test battery administered by trained research assistants in their home language. The neuropsychological test battery was comprised of a number of individual neuropsychological tests previously described in detail (Hoare et al. 2016; Phillips et al. 2018). We conducted Cronbach’s alpha tests on various combinations of neuropsychological tests to determine the statistical strength of each cognitive domain. Internal consistency was determined among the neuropsychological tests within each domain. The threshold for acceptable internal consistency was a Cronbach’s a at least 0.7, which was found for general intellectual functioning, attention, working memory, visual memory, verbal memory, motor coordination, processing speed, and executive function. Composite cognitive domain scores were calculated by averaging the scores of the tests that comprised each domain, so that a single score for each domain was determined for each participant. Experienced trauma was also assessed using the Childhood Trauma Questionnaire (CTQ) (Bernstein et al. 2003).

### Blood collection and processing

Blood samples were drawn at enrolment and at 36-month follow-up. DNA extraction from blood samples was accomplished using the QIAsymphony DSP DNA Midi kit and protocol. DNA was quantified using BioDrop (Whitehead Scientific, Cape Town, South Africa) and normalised to a concentration of 5–10 ng/ml. All samples were tracked using a Laboratory Information Management System (LIMS) (Freezerworks, Seattle, WA, USA). VL, CD4 ratio, and hsCRP were also assessed.

### DNA methylation

DNA methylation (DNAm) analysis was performed with the Illumina Infinium MethylationEPIC BeadChip (Illumina, San Diego, CA, USA), which measures bisulfite conversion-based, single-CpG resolution DNAm levels at 866 836 CpG sites in the human genome. The standard protocol of Illumina methylation assays quantifies methylation levels by the *b* value using the ratio of intensities between methylated (signal A) and unmethylated (signal B) alleles. Specifically, the *b* value is calculated from the intensity of the methylated (M corresponding to signal A) and unmethylated (U corresponding to signal B) alleles, as the ratio of fluorescent signals b¼Max (M,0)/[Max (M,0)þMax (U,0)þ100]. Thus, *b* values range from 0 (completely unmethylated) to 1 (completely methylated) [18]. We used the noob normalisation method (Triche et al. 2013), which is implemented in the ‘minfi’ R package (https://www.r-project.org/) (Aryee et al. 2014).

### DNA methylation age and the epigenetic clock

We used the multi-tissue DNAm age estimator from Horvath (2013), which is defined as a prediction method of age based on the DNAm levels of 353 CpGs. Predicted age, referred to as DNAmAge, correlates with chronological age in sorted cell types (CD4þ T cells, monocytes, B cells, glial cells, neurons), tissues, and organs, including the whole blood, brain, breast, kidney, liver, lung, and saliva (Horvath 2013). Here, we focus on two measures of epigenetic age acceleration denoted by age acceleration difference (AAD) and extrinsic epigenetic age acceleration (EEAA), respectively. Both measures are independent of chronological age (at the time of blood draw). For both measures of age acceleration, positive values indicate that the blood sample is older than expected based on chronological age. AAD is defined as the difference between DNAmAge and chronological age. EEAA can be interpreted as an enhanced version of the Hannum measure of DNAm age estimator, because it up-weights the contributions of age-related blood cell counts (Chen et al. 2016; Horvath et al. 2016). Both AAD and EEAA were computed using the epigenetic clock software (https://dnamage.genetics.ucla.edu/) in which they are denoted as AgeAccelerationDifference and BioAge4HAStaticAdjAge, respectively. Although AAD is relatively robust with respect to changes in blood cell composition, EEAA capitalises on age-related changes in blood cell types and captures aspects of immunosenescence (Chen et al. 2016). The epigenetic clock method and software apply to data generated using any Illumina platform (including the EPIC array). Missing CpG probes were automatically imputed by the software. Mathematical details and software tutorials for the epigenetic clock can be found in the additional files of Horvath (2013). An online age calculator can be found at our webpage: http://labs.genetics.ucla.edu/horvath/dnamage/. For more detail on the calculation and use of these variables, see Horvath et al. (2018). Additionally, a DNAmAge Change score was calculated by subtracting follow-up DNAmAge from baseline DNAmAge for each participant.

### Neuroimaging acquisition

#### Image acquisition

Structural and diffusion-weighted imaging was performed at the Cape Universities Brain Imaging Centre on a 3 T Siemens Allegra scanner (Hoare et al. 2018) with a 16 channel transmit–receive head coil was used to acquire three diffusion-weighted images, each volume having the following parameters: 30 diffusion directions with *b* = 1000 s/mm^2^; repetition time (TR) = 8800 ms; echo time (TE) = 88 ms; in-plane resolution of 2 × 2 mm^2^; and slice thickness of 2.2 mm. A single unweighted volume (*b* = 0 s/mm^2^) was also acquired. The acquisition was repeated 3 times to allow for redundancy in data. Each DTI scan took 5 min (15 min for 3 scans). A multiecho MPRAGE T1-weighted image was acquired with the following parameters: FOV = 256 × 256 mm, TR = 2530 ms, TE = 1.53/3.21/4.89/6.57 ms, TI = 1100 ms, flip angle = 7°, 144 slices, in-plane resolution = 1.3 × 1.0mm2 and slice thickness of 1.0 mm. MPRAGE scan time was 7 min.

#### DTI pre-processing

Diffusion-weighted images were corrected for eddy current distortion within FSL 5.0.1 and imported into MATLABR 2018b for processing. This entailed the affine registration to the average *b* = 0 m/s2 image of the first acquisition. For each of the acquisitions, outlier data points were determined by calculating the *Z* values at the 25th and 75th percentile of the registered diffusion image. Any data points that were 3 SD from the mean were excluded. The corrected images were exported to FSL 5.0.1 after correction. In FSL 5.0.1 images underwent BET to remove any non-brain tissue and fit a linear tensor model to produce fractional anisotropy (FA) and mean diffusivity (MD) maps. FA images were analysed with the TBSS pipeline (Smith et al. 2006). Each participant’s FA was registered to a study-specific target. This target was determined by registering each participant to every other participant. The mean square displacement coefficient of each image was calculated, and the participant with the lowest mean displacement was chosen as a representative target for the group. After registration to the study-specific target, each image was then up-sampled to MNI space, taking into account the previous transformation parameters. An average FA was created and thinned to produce a mean FA skeleton with a threshold of 0.2. This skeleton is representative of the centres of white matter tracts common to the group. Registration and skeleton projection were also applied to the MD, images as described above.

#### Freesurfer pre-processing

T1-weighted images were processed with Freesurfer V5.3 on the Lengau cluster at the Centre for High Performance Computing (CHPC), Rosebank, Cape Town, South Africa. The pipeline has been described previously (Desikan et al. 2006). T1-weighted images were normalised, bias field-corrected, and skull-stripped. Inner and outer cortical surfaces were modelled as triangular tessellation. Cortical thickness measurements were obtained by calculating the distance (in mm) between pial and grey-white matter surfaces at each vertex location (Fischl and Dale 2000). Cortical surface area was calculated as the average of the grey matter vertices over regions. The vertex data was normalised to the ‘fsaverage’ template included with Freesurfer by utilising a curvature matching technique (Fischl et al. 2002). For volumetric data, the brain was segmented into volume-based labels utilising probabilistic methods (Fischl et al. 2002). After reconstruction, each individual scan was checked for any major errors in segmentation, corrected and rerun if needed.

### Statistical analyses

Demographic variables in the PHIV + and HC groups were compared using independent sample *t* tests for continuous variables, and chi-square tests for categorical variables. Independent *t* tests also compared cognitive domain scores between the PHIV + and HC groups (see Table 1).

**Table 1:** Thirty-six-month follow-up demographics and cognitive domains in PHIV + and HC groups

		PHIV +	HC	*t* test or chi-square value	*p* value
		*n* = 60	*n* = 36		
Age in years	Mean (SD)	13.8(0.9)	13.5(0.9)	1.75	0.083
Sex	Male/female	28/32	18/18	0.10	0.752
Ethnicity	Black African/other	58/2	36/0	1.23	0.542
Home language	isiXhosa/other	58/2	35/1	2.87	0.238
Current school grade	Mean (SD)	6.7(1.5)	7.4(1.5)	-2.23	0.028*
Repeated a school grade	No/yes	32/28	18/18	0.10	0.752
Household income bracket^a^	Mean (SD)	3.1(0.67)	3.1(0.67)	0.04	0.969
CTQ score	Mean (SD)	45.1(7.4)	44.3(4.9; *n* = 13)	0.345	0.731
ART regimen	First/second/third/unknown	40/16/2/2	n/a	n/a	n/a
Age of ART initiation	Mean (SD)	3.0(2.3)	n/a	n/a	n/a
Years on ART	Mean (SD)	10.8(1.6)	n/a	n/a	n/a
Viral Load copies/mL	% with values > 40	32%	n/a	n/a	n/a
CD4 T cell count	Mean (SD)	747.5(290.6)	n/a	n/a	n/a
CD4%	%	0.1019	0.1348	2.51	0.014*
hsCRP	Mean (SD)	4.33(7.7)	1.20(2.8)	− 2.77	0.007*
Cognitive domain		*Z* score mean (SD)	*Z* score mean (SD)	*t* test value	*p* value
General intelligence		− 0.52(0.81)	0.000(0.70)	− 3.16	0.002*
Working memory		− 0.78(0.71)	− 0.000(0.58)	− 5.53	< 0.001*
Executive function		− 0.70(0.73)	− 0.000(0.53)	− 4.94	< 0.001*
Language		− 0.15(1.04)	0.002(1.00)	− 0.07	0.486
Motor coordination		− 0.48(0.93)	− 0.000(0.90)	− 2.44	0.017*
Processing speed		− 0.63(0.71)	− 0.000(0.62)	− 4.36	< 0.001*
Attention		− 1.08(1.01)	0.000(0.80)	− 5.39	< 0.001*
Verbal memory		− 0.67(1.57)	0.000(0.78)	− 2.37	0.020*
Visual spatial acuity		0.51(1.45)	− 0.000(1.00)	1.82	0.073
Visual memory		0.17(1.16)	0.000(1.00)	0.073	0.465

*PHIV* perinatally infected HIV +, *HC* healthy control, *CD4* cluster of differentiation 4 cell, *CTQ* childhood trauma questionnaire, *ART* antiretroviral treatment, *hsCRP* highly sensitive C-reactive protein, *SD* standard deviation

*significant beyond a *p* value of 0.05

^a^Household annual income brackets: 1:$0 2:$1–$350 3:$351–$1500 4: > $1500

DNAmAges and the two measures of epigenetic ageing (EEAA and AAD) were compared using *t* tests. This comparison was done between the PHIV + and HC groups with independent sample *t* tests and between baseline and follow-up time points using paired *t* tests. The change in chronological age and change in DNAmAge were also tracked between baseline and follow-up in both groups (see Table 2). Within the PHIV + group, CD4 and VL values were also compared between baseline and follow-up (see results in-text).

**Table 2:** Ages and DNAmAges of PHIV + and HC groups at baseline and 36-month follow-up

	Baseline				Follow-up			
	PHIV +	HC	*t* score	*p* value	PHIV +	HC	*t* score	*p* value
	*n* = 60	*n* = 36			*n* = 60	*n* = 36		
Mean age (SD)	10.8 (0.9)	10.7 (1.0)	− 0.5	0.645	13.8 (0.9)	13.5 (0.9)	− 1.8	0.083
Age range	9.0–12.8	9.0–13.1	n/a	n/a	12.1–15.1	12.1–15.1	n/a	n/a
Mean DNAmAge (SD)	16.1 (3.5)	13.8 (2.9)	− 3.9	< 0.001*	18.8 (5.1)	16.4 (3.7)	− 2.5	0.015*
DNAmAge range	9.5–30.4	9.0–20.9	n/a	n/a	10.3–33.6	10.1–24.8	n/a	n/a
Mean AAD (SD)	5.3 (3.4)	3.1 (2.5)	− 4.0	< 0.001*	5 (4.8)	2.9 (3.2)	− 2.3	0.023*
AAD range	− 2.1–19.4	− 1.5–10.3	n/a	n/a	− 2.8–19.1	− 2.5–10.5	n/a	n/a
Mean EEAA (SD)	0.55 (6.6)	− 2.65 (3.9)	− 4.24	0.002*	1.68 (7.3)	− 2.80 (4.2)	− 3.8	0.001*
EEAA range	− 15–25.7	− 11.7–5.3	n/a	n/a	− 12.1–20.8	− 15–3.2	n/a	n/a

*PHIV* perinatally infected HIV +, *HC* healthy control, *DNAmAge* DNA methylation age, *EEAA* extrinsic epigenetic accelerated ageing, *AAD* age acceleration difference, *SD* standard deviation, *significant beyond a *p* value of 0.05

Similar to our baseline publication, univariate general linear models were constructed with the two follow-up epigenetic ageing acceleration measures as the dependent variables and CD4 ratio, VL category, ART line, highest school year, repeated a grade (yes/no), sex, and chronological age as the independent variables. For these models, the relevant baseline epigenetic ageing score (EEAA or AAD) was also included in the model as a covariate, in order to control for individual starting points (see Table 3).

**Table 3:** Multivariable linear models showing variables associated with epigenetic ageing measures, controlling for baseline values, in the PHIV group. *n* = 88

	Follow-up EEAA. R^2^ = 0.674 (adj = 0.631)			Follow-up AAD. *R*^2^ = 0.588 (adj = 0.535)		
Covariate	Beta coefficient	95% CI	*p* value	Beta coefficient	95% CI	*p* value
Baseline EEAA or AAD score	0.46	0.28–0.64	< 0.001*	0.84	0.61–1.10	< 0.001*
Age	− 0.80	− 1.90–0.31	0.157	0.98	0.16–1.81	0.020*
CD4 ratio	− 46.57	− 62.66– − 30.47	< 0.001*	− 17.69	− 28.77– − 6.62	0.002*
Current grade	− 0.43	− 1.12–0.26	0.214	− 0.37	− 0.87–0.13	0.142
Sex: male vs female	0.48	− 1.45–2.41	0.622	− 0.11	− 1.50–1.29	0.880
VL cat = 0^b^ (HC)	− 4.58	− 11.17–2.01	0.170	0.85	− 3.91–5.62	0.722
VL cat = 1^b^ (PHIV + VL < 40)	− 3.22	− 5.71– − 0.72	0.012*	− 0.52	− 2.35–1.31	0.570
ART line = 1^a^	− 1.96	− 8.16–4.25	0.531	0.019	− 4.45–4.48	0.993
ART line = 2^a^	0.13	− 6.51–6.24	0.967	− 0.21	− 4.77–4.36	0.929
Repeated a grade: no vs yes	1.28	− 0.85–3.42	0.235	1.04	− 0.53–2.61	0.190

*PHIV* perinatally infected HIV +, *HC* healthy control, *DNAmAge* DNA methylation age, *EEAA* extrinsic epigenetic accelerated ageing, *AAD* age acceleration difference, *VL* viral load, *ART* antiretroviral therapy, *SD* standard deviation, *adj* adjusted5

*significant beyond a p value of 0.05

^a^reference category HC

^b^reference category VL > 40

Differences in six mean brain structural values (mean FA, mean MD, subcortical grey matter volume, total grey matter volume, cortical white matter volume, cortical thickness) were assessed between the PHIV + and HC groups, considering both baseline and follow-up values (see Table 4).

**Table 4:** Brain volumes, cortical thickness, and diffusion values of PHIV + and HC groups at baseline and 36-month follow-up

	Baseline mean (SD)				Follow-up mean (SD)			
	PHIV +	HC	*t* score	*p* value	PHIV +	HC	*t* score	*p* value
	*n* = 56	*n* = 31			*n* = 56	*n* = 31		
Cortical white matter volume	363,430 (59,691)	381,628 (52,742)	1.42	0.160	380,766 (78,762)	400,711 (61,560)	1.22	0.227
Subcortical grey matter volume	56,892 (6320)	60,401 (5284)	2.62	0.010*	57,350 (5790)	58,358 (6912)	0.725	0.470
Total grey matter volume	640,282 (89,748)	664,094 (67,061)	1.29	0.201	620,677 (90,384)	641,502 (80,599)	1.07	0.288
Cortical thickness	2.652 (0.142)	2.661 (0.108)	0.32	0.752	2.583 (0.120)	2.586 (0.120)	0.10	0.919
Average FA	0.297 (0.030)	0.300 (0.021)	0.86	0.396	0.426 (0.019)	0.436 (0.014)	1.99	0.051*
Average MD	0.000847 (0.0000296)	0.000851 (0.0000548)	− 0.26	0.794	0.000793 (0.0000326)	0.000776 (0.0000342)	− -1.64	0.105

Sample size differs in this analysis as DTI data were not available for the full sample after DTI quality control checking. Volume brain values are in mm^3+^. *PHIV* perinatally infected HIV + ; *HC* healthy control; *DTI* diffusion tensor imaging; *FA* fractional anisotropy; *MD* mean diffusion; *SD* standard deviation; *significant beyond a *p* value of 0.05

The same six global brain values were then correlated with both EEAA and AAD, both baseline and follow-up values, to detect associations (see Table 5).

**Table 5:** Correlating epigenetic ageing with global brain structural values in the PHIV + group (*n* = 60)

	Baseline EEAA	Follow-up EEAA	Baseline AAD	Follow-up AAD
Cortical white matter volume	− 0.041(0.755)	0.195 (0.136)	− 0.046(0.732)	− 0.001(0.996)
Subcortical grey matter volume	− 0.044(0.743)	0.210 (0.107)	− 0.011(0.935)	0.140(0.285)
Total grey matter volume	− 0.051(0.704)	0.264 (0.041)*	− 0.048(0.720)	0.074(0.574)
Cortical thickness	− 0.123(0.249)	0.044(0.670)	− 0.109(0.307)	− 0.020(0.845)
Average fractional anisotropy (FA)	− 0.281(0.053)	− 0.304(0.034)*	− 0.224(0.125)	− 0.167(0.253)
Average mean diffusion (MD)	0.413(0.003)*	0.294(0.041)*	0.363(0.011)*	0.267(0.063)

Values presented are Pearson’s *r* (*p* value). *indicates significance beyond a *p* value of 0.05

*PHIV* perinatally infected HIV +, *EEAA* extrinsic epigenetic accelerated ageing, *AAD* age acceleration difference, *FA* fractional anisotropy, *MD* mean diffusion

Using SPSS28 (IBM Corp 2021), bivariate correlations were run to detect associations between the six brain structural values and the 10 cognitive domain functioning scores (see results in-text).

Measures of accelerated epigenetic ageing were correlated with performance on 10 domains of cognitive functioning (general intelligence (GI), executive function (EF), working memory (WM), attention (ATT), visual memory (VisM), verbal memory (VM), language (LANG), motor coordination (MC), processing speed (PS), visual spatial acuity (VSA) (see results in-text).

In each test, all participants with the relevant data were included. Due to some missing data from the methylation testing output or from DTI extraction due to scan quality, sample size may differ slightly between all the detailed tests. Sample sizes are provided for each test in the Results section.

## Results

### Demographics

The PHIV + and HC groups were well characterised in terms of age, sex, ethnicity, home language, household income, and childhood trauma. The PHIV + participants were overall one year behind in schooling compared to the HC group. The PHIV + group had a lower CD4 ratio than the HC group. In seven of the ten tested cognitive domains, the PHIV + group performed significantly worse. See Table 1 for details.

### Progression of epigenetic ageing from baseline to 36-month follow-up

At both baseline and follow-up, DNAmAge, EEAA, and AAD were all higher in the PHIV + group. At baseline, the PHIV + group had an average chronological age of 10.8 (SD = 0.9) and a DNAmAge of 16.1 (3.5), while the HC group had an average chronological age of 10.7 (1.0) and a DNAmAge of 13.8 (2.9). Despite having well-matched chronological ages, the PHIV + group had a DNAmAge 2.3 years older than the HC group. At follow-up, the PHIV + and HC groups were still well-matched on chronological age (PHIV +  = 13.8, SD = 0.9, and HC = 13.5, SD = 0.9), and the PHIV + group had a DNAmAge 2.4 years older than the HC group (PHIV +  = 18.8 and HC = 16.4). See Table 2 for details.

While DNAmAge remains higher than chronological age at baseline and follow-up, in the PHIV + group, differences in AAD persisted over 3 years (*t* = 0.18, *p* = 0.86, *n* = 59). Baseline AAD was 5.20 (3.23), and follow-up AAD was 5.11,(4.77). EEAA also did not change significantly (*t* = 1.07, *p* = 0.29, *n* = 59), with baseline EEAA of 0.92 (6.33) and follow-up EEAA of 1.82 (7.32).

In the HC group, AAD and EEAA also did not differ between baseline and follow-up. Baseline AAD was 2.81 (2.39), and follow-up AAD was 3.25 (3.59) (*t* =  − 1.12, *p* = 0.27, *n* = 31). Baseline EEAA was − 2.83 (3.57), and follow-up EEAA was − 2.67 (4.35) (*t* =  − 0.28, *p* = 0.78, *n* = 31).

In the PHIV + group, CD4 counts decreased from a mean of 837 (SD = 351) at baseline to a mean of 753 (SD = 290) at 36-month follow-up. This is a significant decrease (*t* = 2.296, *p* = 0.025). At baseline, 20% of this subsample of the PHIV + group had VL over 40 copies/mL, while at follow-up, 32% of PHIV + group had VL scores over 40 copies/mL.

**Fig. 1:**
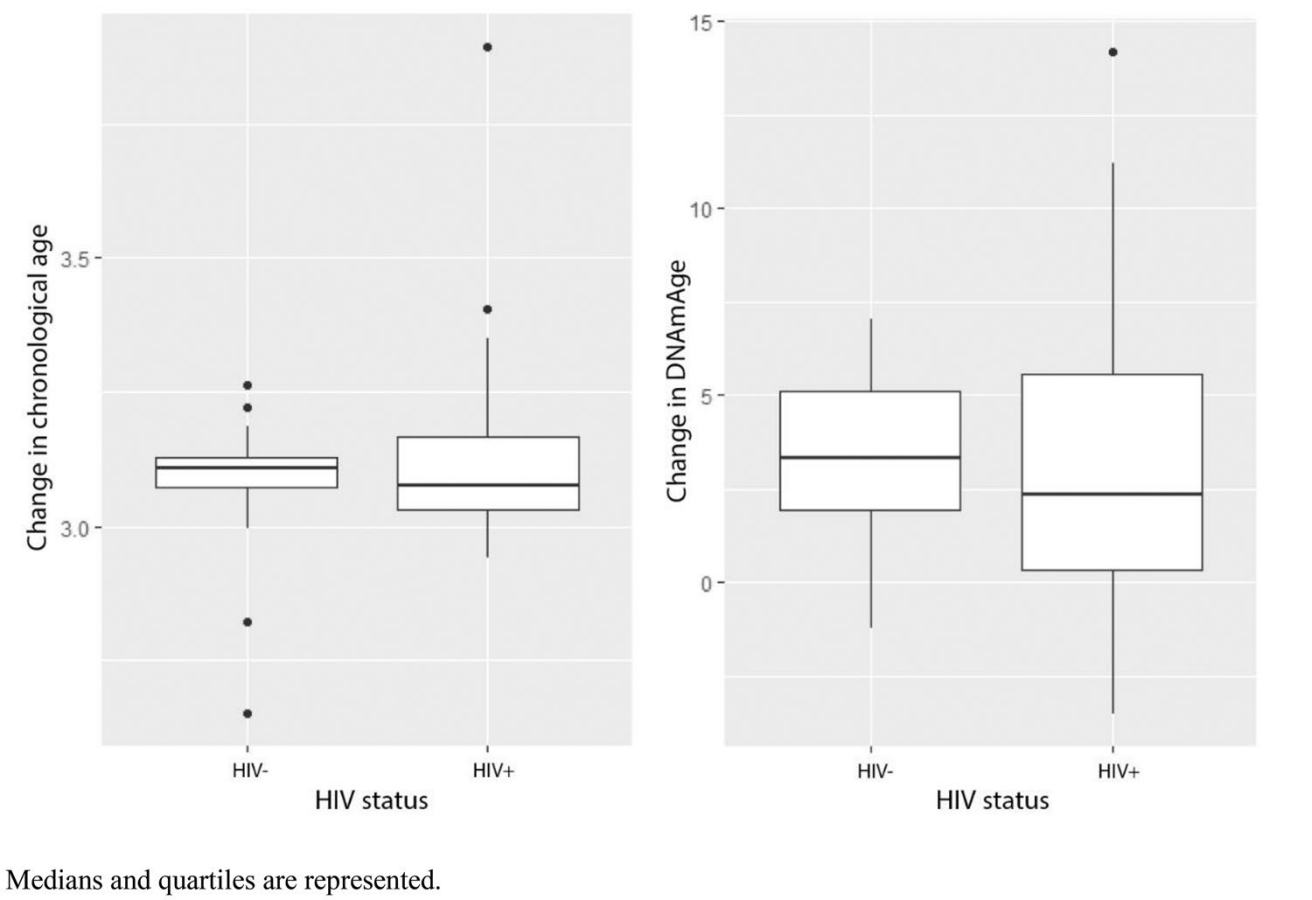
Change in chronological age from baseline to follow-up and change in DNAmAge from baseline to follow-up. Medians and quartiles are represented

In both groups combined, the difference in chronological age between baseline and follow-up was on average 3.11 years (SD = 0.14), and the change in DNAmAge was 3.05 years (SD = 3.25). This variance in chronological age and in DNAmAge is visualised for both the HC and PHIV + groups in Fig. 1. The change in DNAmAge included considerably more variance (PHIV + : mean = 2.88, SD = 3.36; HC: mean = 3.36, SD = 2.21) than the change in chronological age (PHIV + : mean = 3.11 SD = 0.15; HC: mean = 3.09, SD = 0.11).

### Virologic, treatment, and education variables associated with follow-up epigenetic ageing

Multivariable linear models showed that a lower CD4 ratio and VL are significantly associated with increased EEAA, while lower CD4 ratio and higher age are significantly associated with increased AAD. All of these measures were taken at follow-up, apart from baseline EEAA and baseline AAD scores. See Table 3.

### Comparing baseline and follow-up brain structural values

Structural whole brain measures of the brain were compared between the PHIV + and HC group at both baseline and follow-up. Subcortical grey matter volumes were higher in the HC group at baseline but not at follow-up. FA values were higher in the HC group at follow-up (*p* = 0.051). See Table 4 for details.

### Associations between brain structural values and epigenetic ageing

Follow-up EEAA was positively associated with total grey matter and lower FA. A consistent finding from these tests was that epigenetic ageing (both EEAA and AAD) is positively associated with MD, a measure of central nervous system (CNS) inflammation. See Table 5 for details.

### Epigenetic ageing and cognitive functioning

In the PHIV + group, chronological age at follow-up correlated negatively with multiple cognitive domain scores (GIF, WM, EF, ATT, PS). In contrast, a positive relationship between cognitive functioning scores and chronological age was present in the HC group for two cognitive domains: language (*r* = 0.42, *p* = 0.011) and verbal memory (*r* = 0.34, *p* = 0.043).

In the PHIV + group (*n* = 60), none of the cognitive functioning domains correlated with follow-up AAD or EEAA values. In the HC group (n = 35), follow-up AAD was negatively associated with executive function (*r* =  − 0.39, *p* = 0.019) and processing speed (*r* =  − 0.34, *p* = 0.046). Follow-up EEAA was positively associated with motor coordination (*r* = 0.40, *p* = 0.017). As a sensitivity analysis, these tests were rerun using only the PHIV + and HC participants with EEAA worse than the median score. In this smaller PHIV + group (*n* = 30), higher AAD was associated with worse motor coordination (*r* =  −0.42, *p* = 0.021). In the smaller HC group (*n* = 18), higher EEAA was associated with poorer performance in general intelligence (*r* =  − 0.51, *p* = 0.035), executive function (*r* =  − 0.51, *p* = 0.039), and attention (*r* =  − 0.52, *p* = 0.033). Higher AAD was associated with poorer executive function (*r* =  −0.50, *p* = 0.044).

### Associations between follow-up brain structure and follow-up cognitive functioning

In the PHIV + group (*n* = 60), whole brain FA was significantly correlated with cognitive functioning. Specifically, improved working memory (*r* = 0.284, *p* = 0.048) and processing speed (*r* = 0.321, *p* = 0.024) scores were associated with higher FA values.

In the HC group (*n* = 35), whole brain MD was significantly correlated with cognitive functioning. Specifically, MD scores were negatively correlated with 5 of the 10 cognitive domains: GI (*r* =  − 0.37, *p* = 0.050), WM (*r* =  − 0.44, *p* = 0.019), EF (*r* =  − 0.42, *p* = 0.026), VSA (*r* =  − 0.41, *p* = 0.029), and VisM (*r* =  − 0.43, *p* = 0.024). The whole brain volumes, thickness, and FA scores did not correlate with any cognitive domain scores.

## Discussion

Overall, the PHIV + and HC groups were well-matched demographically; however, school progression and cognitive performance differed significantly, with the PHIV + one school year behind controls, and performing worse than the controls on seven out of ten cognitive domains.

DNAmAge and accelerated epigenetic ageing remained higher in PHIV + compared to HC, with the differences between groups staying similar from baseline to follow-up. That is, there was no further divergence between the groups in age acceleration. The change in DNAmAge over that time period however was quite varied, with a large standard deviation, which indicates that in some participants, DNAmAge has sped up or slowed down substantially. This may be due to changing behaviours in ART adherence and fluctuations of metabolic health throughout adolescence in our sample. At follow-up, the difference in epigenetic ageing between the two groups was still significant, with the PHIV + having advanced ageing, but that difference had narrowed over the three-year period between data collection points.

At this follow-up time point, ART line was no longer associated with increased epigenetic ageing variables. While in our sample the main reason for first-line failure is poor adherence and side effects, studies have shown that ART toxicity in adolescents on first- and second-line treatments has been linked to oxidative DNA damage and protein carbonyl content and associated increased ageing in HIV patients (Kolgiri and Patil 2017). A lower CD4 ratio remained associated with accelerated ageing, and we showed that accelerated epigenetic ageing is still most severe in PHIV + with detectable VL, indicating a persistent relationship between immune function and ageing in PHIV. This finding is consistent with findings in adult American males on ART (Horvath and Levine 2015) as well as our baseline study in children aged 9–12 (Hoare et al. 2018). Some genes underlying general cellular immune response and inflammation, such as NLRC5, have shown differential methylation in HIV infection, suggesting that epigenetic modification of relevant immunity-regulating genes is present in HIV (Shiau et al. 2019; Yang et al. 2020).

The neuroimaging data showed that global DTI measures were associated with accelerated epigenetic ageing in the PHIV + group, specifically increased MD and decreased FA. This is consistent with a previous finding in a subsample of our broader healthy cohort in which DTI scalars emerge as being particularly sensitive to CNS changes associated with epigenetic ageing and HIV infection (Hoare et al. 2020, 2021). MD is a measure of water molecule mobility in white matter tissue while FA measures white matter integrity. As such, lower FA and higher MD indicate damage to white matter microstructure. Children aged 5 and 7 have previously shown disrupted white matter development in multiple regions with lower FA values and higher MD values, regardless of ART initiation date (Jankiewicz et al. 2017) although interrupted ART may worsen this effect (Ackermann et al. 2016). Another study in slightly older HIV + children (6–15 years) also found decreased FA and increased MD, but this effect was associated with being on second-line ART, as well as increased VL and lower albumin and haemoglobin (Hoare et al. 2015a, b). Adolescent studies have shown this same pattern of lower FA and increased MD, along with poorer neuropsychological performance (Hoare et al. 2015b).

Adolescence is a critical period of change in brain volume. Although cortical white matter volume increased across both of our groups, there were differences in the overall neurodevelopment of grey matter volume. While the control group saw decreases in both subcortical and total grey matter volumes, as is expected in this age group (Sowell et al. 2003), the PHIV + group had an increase in subcortical grey matter volume. It is thought that this may be indicative of a disruption to the intensive synaptic pruning of unnecessary neural connections that occurs during adolescence (Squeglia et al. 2009). This thinking is in line with the finding of Sarma et al. (2014) who found greater volumes in temporal, frontal, occipital, and subcortical grey matter regions in PHIV + adolescents (mean age = 17, SD = 2.9) compared to controls, although they did not detect a global increase in grey matter volume. They proposed neuronal cell swelling as another possibility for this finding and that these mechanisms may be affected by wither HIV infection of ART medications.

Contrary to our baseline study (Horvath et al. 2018), cognitive functioning was only associated with accelerated ageing in the HC group. This may be due to a smaller sample size being used for the follow-up analyses. However, in the HC group, executive function and processing speed were worse in those with increased rates of epigenetic ageing. Notably, the relationship between cognitive functioning and chronological age is concerning within the PHIV + group as there was a negative association between chronological age and cognitive performance at both baseline and follow-up. Cognitive function is expected to improve with chronological age as in the HC group in the current study. Few studies have tracked cognitive changes over time in HIV + youth, although one study with two time points showed three of the eleven child participants dropping in relative cognitive function over time, with inconclusive findings (Gosling et al. 2004). Another study found stable cognitive function but declining language skills in children 24 months after starting ART (Wolters et al. 1997). HIV can directly affect neurodevelopment, with cognitive performance being modulated by the effects of HIV on the CNS. HIV could also affect cognition and neurophysiology by more explicitly accelerating the ageing process, as shown in studies of HIV + adults (Aberg 2012; Cole et al. 2017). In many studies, PHIV + have worse cognitive function compared to their HIV-uninfected peers, across multiple cognitive domains (Phillips et al. 2016; Ruel et al. 2012; Willen 2006).

In non-HIV studies, variations in epigenetic ageing have been detected in different ethnicities from North and South America. In adults, African Americans have consistently lower epigenetic ageing (intrinsic or extrinsic EAA) than European Americans, across two cohort studies (Horvath et al. 2016, examining multiple cohorts). Additionally, men have higher epigenetic ageing than women, particularly in Hispanic and Caucasian groups. This sex difference may be related to blood cell count-related rapid immunosenescence and lower CD4 T cell count (Horvath et al. 2016). No epigenetic ageing differences were detected between ethnicities in child/young adult cohorts. Similarly, in a study of British children below 8 years old (97% Caucasian), there were no epigenetic ageing differences between boys and girls according to the Horvath epigenetic ageing clock (Marini et al. 2020). This is in line with our findings and suggests that sex-based differences in epigenetic ageing may only develop to detectable levels later in life.

Limitations of this study include a small sample size, which was due to the expense of laboratory investigations of neuroimaging and DNA methylation testing. In addition, although we employ extensive screening and assessment protocols, undiagnosed infections or other conditions may have been present, moderating numerous blood values. ART adherence is only measured on a self-report basis. ART line is important to note (as reported in Tables 1 and 3); however, the basis on which an ART line fails is often not known.

## Conclusion

In summary, we found that PHIV + adolescents show persistent epigenetic ageing at a follow-up time point when compared to well-matched controls. Increased variance in DNAmAging might indicate varying degrees of immune health response to treatment and development trajectories. Accelerated epigenetic ageing is also associated with virologic and treatment-related variables such as CD4 ratio and VL, indicating the importance of disease management during the adolescent stage. Neuroimaging results showed that epigenetic ageing is associated with reduced FA and increased MD, indicating white matter microstructural damage. PHIV + additionally have larger grey matter volumes, possibly due to ineffective pruning during the adolescent period.

## Data Availability

The data is available upon request.
